# Diagnostic value of DNA or RNA-based metagenomic next-generation sequencing in lower respiratory tract infections

**DOI:** 10.1016/j.heliyon.2024.e30712

**Published:** 2024-05-03

**Authors:** Jiafu Song, Suxia Liu, Yongpeng Xie, Chen Zhang, Caiyun Xu

**Affiliations:** a Department of Respiratory and Critical Care Medicine, The Affiliated Lianyungang Hospital of Xuzhou Medical University, Lianyungang, Jiangsu, China; b Department of Critical Care Medicine, The Affiliated Lianyungang Hospital of Xuzhou Medical University, Lianyungang, Jiangsu, China; c Department of Medicine, Dinfectome Inc., Nanjing, Jiangsu, China

**Keywords:** Bacterial pneumonia, Fungal pneumonia, Tuberculosis, RNA-mNGS, DNA-mNGS

## Abstract

**Objectives:**

We aimed to evaluate and compare the diagnostic performance of RNA-mNGS and DNA-mNGS workflow in bacterial pneumonia, fungal pneumonia and tuberculosis.

**Methods:**

A total of 134 cases suspected pneumonia undergoing both DNA and RNA based mNGS of bronchoalveolar lavage fluid (BALF) and also traditional etiological examination were evaluated retrospectively.Sensitivity, specificity, PPV, NPV and accuracy rate of DNA and RNA based mNGS were estimated.

**Results:**

In the diagnosis performance of bacterial pathogens in LRTIs,the specificity of RNA-mNGS was higher than that of DNA-mNGS(82.3 % vs. 61.9 %, *P* < 0.01). There was no significant difference of sensitivity between the two process(71.4 % vs. 85.7 %, *P* = 0.375).In the diagnosis performance of fungal pathogens in LRTIs,the specificity of RNA-mNGS was higher than that of DNA-mNGS (72.3 % vs. 27.3 %,*p* < 0.001). There was no significant difference of sensitivity between the two process(96.5 % vs. 98.8 %,*p* = 0.125).In the diagnosis performance of tuberculosis in LRTIs,the sensitivity of DNA-mNGS was higher than that of RNA-mNGS (91.7 % vs. 33.3 %,*p* = 0.016),the specificity was similar in the two process (100 %).

**Conclusions:**

RNA-mNGS may reduced the misdiagnosis rate of bacterial and fungal pathogens in LRTIs.Compared to RNA-mNGS, DNA-mNGS may could improve the diagnostic rate of tuberculosis.

Compared with conventional culture, metagenomic next-generation sequencing (mNGS) is an unbiased approach to detect all pathogens in a clinical sample and has high sensitivity and short turnaround time. However, there was a low specificity due to bacterial colonization and pollution of the lower respiratory tract [1].Therefore interpretation of mNGS results is a huge challenge. Investigators are committed to searching for unifified standard on how to interpret mNGS indicators such as sequencing reads, genomic coverage, and relative abundance of each organism to distinguish between colonizing strains and actual pathogens [2]. However, we could not precisely assess the numbers of bacterial colonization, and numbers of questionable pathogenic bacteria may larger than pathogenic bacteria, that may cause misjudgment. Due to the cost of medical economics and DNA composes the genetic material for pathogenic bacteria and fungi, mNGS based RNA and DNA is rarely simultaneous used for detection of pathogenic microorganisms in clinical diagnosis unless RNA viruses are suspected. Based on the theory that the responsible microorganism may be more active and produce more transcripts than the colonized microorganism, and the transcripts of DNA organisms can also be detected through the RNA workflow, this study was performed to evaluate and compare the diagnostic performance of RNA-mNGS and DNA-mNGS workflow in lower respiratory tract infections (LRTIs).

## 1 Method

### 1.1 Participants

This retrospective study included a total of 134 patients with suspected LRTIs who were admitted to the First Affiliated Lianyungang Hospital of Xuzhou Medical University from January 2021 to December 2023. Patients were enrolled according to the Guidelines for the management of adult lower respiratory tract infections of the European Respiratory Society and European Society for Clinical Microbiology and Infectious Diseases [3].In this study, 134 bronchoalveolar lavage fluid (BALF) samples from patients with LRTIs were assessed using mNGS (DNA and RNA) assays.

### 1.2 Specimen collection

Following admission, alveolar lavage was performed on all patients, with 2 portions 10–20 mL each.NGS was used for one case, while traditional etiology was used for the other case. BALF samples were collected according to standard procedures [4]. samples were sent for mNGS(Dinfectome Inc., Nanjing, China).

### 1.3 Conventional microbiological tests

Traditional etiological examination for suspected pneumonia were retrospectively analysed. Data entry was done by two researchers alone and cross-checked to guarantee the accuracy. Clinical infection or non-infection and the results of the pathogenic agents were determined comprehensively through the patient's symptoms, signs, chest x-ray or CT, smear and culture and mNGS by another two experienced specialists, discrepant results were adjudicated by a third expert.

### 1.4 Nucleic acid extraction

Patients' BALF were collected following standard procedures.The DNA was extracted with the TIANamp Bacteria DNA Kit (China, TIANGEN). Nanodrop (Thermo Fisher Scientific) and Qubit (Thermo Fisher Scientific) were used to measure the quality and quantity of the extracted DNA.To extract RNA from supernatants, we used the QIAamp viral RNA mini kit (from QIAGEN).

### 1.5 Library preparation and sequencing

DNA sequencing libraries were prepared using the Hieff NGS C130P2 OnePot II DNA Library Prep Kit for MGI (Yeasen Biotechnology) according to the manufacturer's instructions. RNA was purified, and rRNA was removed from the purified RNA, A purified and amplified library was then constructed. The quality control was performed using 2100 Agilent High Sensitive DNA chips (Agilent),and libraries were sequenced by Illumina HiSeq 2000 on a single-end basis (50 bp).

### 1.6 Sequencing data processing

We use in-house developed bioinformatics pipeline for pathogen identification. Briefly, Adapter contamination, duplicate reads, low-quality reads, and reads shorter than 35 bases were removed from high-quality sequencing data.A bowtie2 mapping to the human reference genome (hg38) excluded human sequences. In order to identify pathogens, reads that were unable to be mapped to human genomes were retained and aligned with microorganism genome database. The microorganism genome database contains genomes of viruses, bacteria, fungi(download from https://www. ncbi. nlm.nih.gov/).

### 1.7 Interpretation and reporting

The mNGS pathogen detection pipeline was described in previous studies [5], and the criteria for detection positivity were as follows: 1) at least one species-specific read for Mycobacterium, Nocardia, and Legionella pneumophila detection; 2) for other virus, bacteria, and fungi, only the reads mapped to sites covered by at least three reads were retained; 3)if the ratio of microorganism reads per million of a given sample versus NTC was<10,the pathogens were excluded.

### 1.8 Statistical analysis

Utilizing the expert's assessment of the pathogen as the gold standard.The true positives (TP) are defined as cases in which pathogens identified by DNA-mNGS or RNA-mNGS are concordant with the gold standard, while false positives (FP) are cases in which pathogens identified by DNA-mNGS or RNA-mNGS do not align with the gold standard. True negatives (TN) refer to instances in which both DNA-mNGS or RNA-mNGS and the gold standard indicate the absence of pathogens, and false negatives (FN) refer to pathogens missed by DNA-mNGS or RNA-mNGS.the sensitivity [TP/(TP + FN)], specifcity [TN/(TN + FP)], PPV[[TP/(TP + FP)], NPV[TN/(TN + FN)],and accuracy rate[(TP + TN)/(TP + FP + TN + FN)] of DNA based and RNA based mNGS were estimated.We used SPSS 26.0 software for statistical analysis (IBM SPSS 26.0, SPSS Inc.).The Kappa statistic and McNemars test were used for tests of sensitivity and specificity agreement of DNA and RNA based mNGS.Statistical significance: *p* value, 0.05 threshold.

## 2 Results

1. Participants' Baselines:

The characteristics are presented in Table 1. A total of 134 adults with suspected LRTIs were enrolled. These 83 males and 51 females with an average age of 63.1 ± 17.8 years,120 cases had LRTIs,14 cases had Non- LRTIs, Of the 120 patients that had LRTIs,90 cases had bacterial infection,23 cases had fungal infection,12 cases had pulmonary tuberculosis, and 34 cases were mixed infections.26 cases had secondary bacterial or fungal infection following virus infection.

2. Evaluating the performance of DNA-mNGS and RNA-mNGS in the diagnosis of bacterial pathogens in LRTIs.

**Table 1:** Baselines characteristics of participants.

Parameters	value
LRTIs (n)	120
Bacterial infection	90
Fungal infection	23
pulmonary tuberculosis	12
Viral infection	35
Mycoplasma pneumoniae	3
mixed infections(n)	34
Non- LRTIs (n)	14
Gender(n)	
Male	83
Female	51
Age, year	63.1 ± 17.8
Underlying disease(n)	85
CAP(n)	103
HAP(n)	17

CAP:community-acquired pneumonia; HAP:hospital-acquired pneumonia.

90 patients were diagnosed with bacterial infections of the LRTIs based on expert opinion. the bacteria with the highest sequencing reads and relative abundance in the RNA and DNA-mNGS process are recognized as agents detected by mNGS. Taking expert judgement as the gold standard for bacterial pneumonia, The sensitivity, specificity, PPV, NPV and accuracy rate of DNA based and RNA based mNGS were compared in Table 2. The specificity of RNA-mNGS was higher than that of DNA-mNGS (82.3 % vs. 61.9 %, *P* < 0.01). There was no significant difference of sensitivity between the two process(71.4 % vs. 85.7 %, *P* = 0.375).

3 Evaluating the performance of DNA-mNGS and RNA-mNGS in the diagnosis of fungal pathogens in LRTIs.

**Table 2:** Diagnostic performance of DNA-mNGS and RNA-mNGS in the diagnosis of bacterial pathogens in LRTIs.

	Se	Sp	PPV	NPV	ACC
DNA-mNGS	85.7	61.9	29.5	95.9	65.7
RNA-mNGS	71.4	82.3	42.9	93.9	80.6
Kappa	Kappa = −0.018 *P* = 0.828	Kappa = 0.126 *P* = 0.163			
McNemer	*P* = 0.375	*P* < 0.01			

Se:sensitivity; Sp:specificity (*Sp*); NPV:negative prediction rate; PPV:positive prediction rate; ACC:accuracy.

Based on expert opinion, 23 patients were diagnosed with or concurrent with fungal pneumonias, the fungus with the highest sequencing reads and relative abundance in the DNA and RNA-mNGS process are recognized as agents detected by mNGS.Taking expert judgement as the gold standard for fungal pneumonias,The sensitivity, specificity, PPV, NPV and accuracy rate of DNA based and RNA based mNGS were compared in Table 3. The specificity of RNA-mNGS was higher than that of DNA-mNGS (72.3 % vs. 27.3 %,*p <* 0.001). There was no significant difference of sensitivity between the two process(96.5 % vs. 98.8 %,*p* = 0.125).

4 Evaluating the performance of DNA-mNGS and RNA-mNGS in diagnosis of tuberculosis.

**Table 3:** Diagnostic performance of DNA-mNGS and RNA-mNGS in the diagnosis of Fungal pathogens in LRTIs.

	Se	Sp	PPV	NPV	ACC
DNA-mNGS	98.8	27.3	73.6	92.3	75.4
RNA-mNGS	96.5	72.3	86.5	91.9	88.1
Kappa	Kappa = 0.511 *P* = 0.005	Kappa = 0.488 *P* < 0.01			
McNemer	*P* = 0.125	*P* < 0.001			

Based on expert opinion, 12 patients were diagnosed with tuberculosis, The mNGS test results have tuberculosis read no matter how many suggested TB infection. Taking expert judgement as the gold standard for TB,the sensitivity, specificity, PPV, NPV and accuracy rate of DNA based and RNA based mNGS were compared in Table 4. The sensitivity of DNA-mNGS was higher than that of RNA-mNGS (91.7 % vs. 33.3 %,*p* = 0.016),the specificity was similar in the two process (100 %).

**Table 4:** Diagnostic performance of DNA-mNGS and RNA-mNGS in the diagnosis of tuberculosis pathogens in LRTIs.

	Se	Sp	PPV	NPV	ACC
DNA-mNGS	91.7	100	100	99.2	99.3
RNA-mNGS	33.3	100	100	93.8	94.0
Kappa	Kappa = 0.087 *P* = 0.460				
McNemer	*P* = 0.016				

## 3 Discussion

Compared to DNA-mNGS,RNA-mNGS reduced the misdiagnosis rate of bacterial pathogens in LRTIs. Clinician are accustomed to the single DNA testing process unless viral infection is suspected [6,7], so both colonization and contamination in BALF will be detected. Thus, the sensitivity of mNGS was much higher [8]but very low specificity than plasma mNGS or traditional microbiological culture [9,10]. However, low specificity of mNGS would lead to judgement errors. It is hypothesized that active microbes may exhibit a higher RNA to DNA ratio compared to dormant microbes. The utilization of RNA-mNGS has been shown to enhance the ratio of microbial sequencing reads, increase read numbers, and improve genomic coverage rates. This, in turn, facilitates the identification of bacteria and aligns with clinical observations [11].A recent study [12] utilized RNA-based mNGS on BALF samples to improve the detection of RNA viruses, DNA viruses, fungi, and bacteria. Our study found that specificity of RNA-mNGS is significantly higher than DNA-mNGS, negative RNA-mNGS results may be used to exclude bacterial and fungal LRTIs. However, the impact of RNA-based sequencing on patient outcomes for LRTIs remains unexplored, for DNA and RNA-mNGS were conducted simultaneously in this study. Future research will involve a comparison of patient outcomes between mNGS tests of DNA alone and DNA combined with RNA.

Compared to RNA-mNGS,DNA-mNGS could improve the diagnostic rate of tuberculosis(TB).Although the incidence of pulmonary tuberculosis (PTB) in mainland China showed a downward trend [13],we still have a moderately high incidence of TB. The diagnosis of TB is difficult in our country for the latent TB infection, active TB and healed TB patients could be detected the antibody. A systematic review included a total of 68,544 patients with PTB found that Xpert MTB/RIF pooled sensitivity ranged between 45.7 % and 73.0 % [14].In this study, the sensitivity of DNA-mNGS for the diagnosis of PTB is better than RNA-mNGS,or traditional etiological examination in previous studies.For patients suspected PTB, traditional pathogen detection fails to yield a diagnostic result,DNA-mNGS is a good choice.Tuberculosis is caused by intracellular bacteria and can be detected following their release from host cells. Specimens obtained from BALF were subjected to mechanical and enzymatic dissociation for DNA extraction in DNA-mNGS. In contrast, RNA extraction for RNA-mNGS involves a gentler cell-wall disruption method, making it challenging to detect tuberculatus. Hence DNA-mNGS demonstrates a significantly higher sensitivity for detecting TB compared to RNA-mNGS.

Our results indicate that predicting the species of pathogens is a crucial consideration for clinicians and researchers when choosing the most suitable sequencing approach for various infectious diseases. RNA-mNGS may reduced the misdiagnosis rate of bacterial and fungal pathogens in LRTIs.Compared to RNA-mNGS, DNA-mNGS may could improve the diagnostic rate of TB.However, mNGS is not currently deemed a suitable replacement for traditional diagnostic tests in the context of LRTIs and is not advised for routine application. NGS assays exhibit promise in enhancing the clinical identification of pathogens in cases where conventional bacterial detection methods prove inadequate. Our findings suggest that in instances where a bacterial or fungal infection is suspected, RNA-mNGS may be utilized independently due to its superior specificity compared to DNA-mNGS, and with no significant disparity observed in the sensitivity between the two sequencing methods.When suspecting TB infection, DNA-mNGS can be performed independently due to the comparable specificity between DNA-mNGS and RNA-mNGS, with the former demonstrating superior sensitivity. In cases where pathogen species cannot be accurately predicted, simultaneous testing of DNA and RNA through mNGS is recommended. If DNA-mNGS detects pathogens with high reads and coverage rates compared to RNA-mNGS, these microorganisms may not be disease-causing and may not require antimicrobial therapy, at least in the short term following specimen collection.

## Clinical trial registration

China Clinical Trial Registration Center, MR-32-24-052497.

## Sources of funding

This study did not receive any form of funding

## Ethical approval

This study was reviewed and approved by the Ethical Review Committee of The Affiliated Lianyungang Hospital of Xuzhou Medical University, with the approval number: LW-20230508001-01.

Informed consent was not required for this was a retrospective review study.

## Data sharing statement

The data that support the findings of this study are available from the corresponding author upon reasonable request.

## CRediT authorship contribution statement

**Jiafu Song:** Methodology, Data curation. **Suxia Liu:** Methodology, Conceptualization. **Yongpeng Xie:** Visualization, Validation, Supervision, Formal analysis. **Chen Zhang:** Methodology. **Caiyun Xu:** Writing – review & editing, Writing – original draft, Resources, Methodology, Investigation.

## Declaration of competing interest

The authors declared no conflicts of interest.

## References

[1] Wang J., Han Y., Feng J. (2019). Metagenomic next-generation sequencing for mixed pulmonary infection diagnosis. BMC Pulm. Med..

[2] Liu H., Zhang Y., Yang J., Liu Y., Chen J. (2022). Application of mNGS in the etiological analysis of lower respiratory tract infections and the prediction of Drug resistance. Microbiol. Spectr..

[3] Woodhead M., Blasi F., Ewig S., Garau J., Huchon G., Ieven M., Ortqvist A., Schaberg T., Torres A., van der Heijden G., Read R., Verheij T.J.M. (2011).

[4] Levy L., Juvet S.C., Boonstra K., Singer L.G., Azad S., Joe B., Cypel M., Keshavjee S., Martinu T. (2018). Sequential broncho-alveolar lavages reflect distinct pulmonary compartments: clinical and research implications in lung transplantation. Respir. Res..

[5] Zeng X., Wu J., Li X., Xiong W., Tang L., Li X., Zhuang J., Yu R., Chen J., Jian X., Lei L. (2022). Application of metagenomic next-generation sequencing in the etiological diagnosis of infective endocarditis during the perioperative period of cardiac surgery: a prospective cohort study. Front Cardiovasc Med.

[6] Zhang P., Chen Y., Li S., Li C., Zhang S., Zheng W., Chen Y., Ma J., Zhang X., Huang Y., Liu S. (2020). Metagenomic next-generation sequencing for the clinical diagnosis and prognosis of acute respiratory distress syndrome caused by severe pneumonia: a retrospective study. PeerJ.

[7] Gu J., Chen L., Zeng C., Yang X., Pan D., Cao H., Qian Q. (2022). A retrospective analysis of metagenomic next generation sequencing (mNGS) of cerebrospinal fluid from patients with suspected encephalitis or meningitis infections. J Healthc Eng.

[8] Huang J., Jiang E., Yang D., Wei J., Zhao M., Feng J., Cao J. (2020). Metagenomic next-generation sequencing versus traditional pathogen detection in the diagnosis of peripheral pulmonary infectious lesions. Infect. Drug Resist..

[9] Sun T., Liu Y., Cai Y., Zhai T., Zhou Y., Yang B., Wu X., Zhan Q. (2022). A paired comparison of plasma and bronchoalveolar lavage fluid for metagenomic next-generation sequencing in critically ill patients with suspected severe pneumonia. Infect. Drug Resist..

[10] He P., Wang J., Ke R., Zhang W., Ning P., Zhang D., Yang X., Shi H., Fang P., Ming Z., Li W., Zhang J., Dong X., Liu Y., Zhou J., Xia H., Yang S. (2022). Comparison of metagenomic next-generation sequencing using cell-free DNA and whole-cell DNA for the diagnoses of pulmonary infections. Front. Cell. Infect. Microbiol..

[11] Zhao N., Cao J., Xu J., Liu B., Liu B., Chen D., Xia B., Chen L., Zhang W., Zhang Y., Zhang X., Duan Z., Wang K., Xie F., Xiao K., Yan W., Xie L., Zhou H., Wang J. (2021). Targeting RNA with next‐ and third‐generation sequencing improves pathogen identification in clinical samples. Adv. Sci..

[12] Shen J., Hu Y., Lv J., Zhao H., Wang B., Yang S., Du A., Liu S., An Y. (2022). Lung microbiota signature and corticosteroid responses in pneumonia-associated acute respiratory distress syndrome in hematological patients. J. Inflamm. Res..

[13] Jiang H., Liu M., Zhang Y., Yin J., Li Z., Zhu C., Li Q., Luo X., Ji T., Zhang J., Yang Y., Wang X., Luo Y., Tao L., Zhang F., Liu X., Li W., Guo X. (2021). Changes in incidence and epidemiological characteristics of pulmonary tuberculosis in mainland China, 2005-2016. JAMA Netw. Open.

[14] Kay A.W., González F.L., Takwoingi Y., Eisenhut M., Detjen A.K., Steingart K.R., Mandalakas A.M. (2020). Xpert MTB/RIF and Xpert MTB/RIF Ultra assays for active tuberculosis and rifampicin resistance in children. Cochrane Db Syst Rev.

